# A highly sensitive and specific workflow for detecting rare copy-number variants from exome sequencing data

**DOI:** 10.1186/s13073-020-0712-0

**Published:** 2020-01-30

**Authors:** Ramakrishnan Rajagopalan, Jill R. Murrell, Minjie Luo, Laura K. Conlin

**Affiliations:** 1 Division of Genomic Diagnostics, Department of Pathology and Laboaratory Medicine, Children’s Hospital of Philadelphia, Philadelphia, PA USA; 2 School of Biomedical Engineering, Science and Health Systems, Drexel University, Philadelphia, PA USA; 3 Department of Pathology and Laboratory Medicine, Perelman School of Medicine, University of Pennsylvania, Philadelphia, PA USA

**Keywords:** Clinical exome sequencing, Copy-number variation

## Abstract

**Background:**

Exome sequencing (ES) is a first-tier diagnostic test for many suspected Mendelian disorders. While it is routine to detect small sequence variants, it is not a standard practice in clinical settings to detect germline copy-number variants (CNVs) from ES data due to several reasons relating to performance. In this work, we comprehensively characterized one of the most sensitive ES-based CNV tools, ExomeDepth, against SNP array, a standard of care test in clinical settings to detect genome-wide CNVs.

**Methods:**

We propose a modified ExomeDepth workflow by excluding exons with low mappability prior to variant calling to drastically reduce the false positives originating from the repetitive regions of the genome, and an iterative variant calling framework to assess the reproducibility. We used a cohort of 307 individuals with clinical ES data and clinical SNP array to estimate the sensitivity and false discovery rate of the CNV detection using exome sequencing. Further, we performed targeted testing of the *STRC* gene in 1972 individuals. To reduce the number of variants for downstream analysis, we performed a large-scale iterative variant calling process with random control cohorts to assess the reproducibility of the CNVs.

**Results:**

The modified workflow presented in this paper reduced the number of total variants identified by one third while retaining a higher sensitivity of 97% and resulted in an improved false discovery rate of 11.4% compared to the default ExomeDepth pipeline. The exclusion of exons with low mappability removes 4.5% of the exons, including a subset of exons (0.6%) in disease-associated genes which are intractable by short-read next-generation sequencing (NGS). Results from the reproducibility analysis showed that the clinically reported variants were reproducible 100% of the time and that the modified workflow can be used to rank variants from high to low confidence. Targeted testing of 30 CNVs identified in *STRC*, a challenging gene to ascertain by NGS, showed a 100% validation rate.

**Conclusions:**

In summary, we introduced a modification to the default ExomeDepth workflow to reduce the false positives originating from the repetitive regions of the genome, created a large-scale iterative variant calling framework for reproducibility, and provided recommendations for implementation in clinical settings.

## Background

Exome sequencing (ES) is a common standard of care diagnostic tool for identifying molecular causes in individuals with suspected Mendelian disorders [1]. Identifying single nucleotide variants (SNVs) and small insertion/deletions (indels) from next-generation sequencing (NGS) data have been well studied and characterized [2, 3]. Success has been elusive to date to detect copy-number variants (CNVs) from NGS data with the same confidence as SNVs/indels. Chromosomal microarrays (CMA), including both array CGH and SNP arrays, are still the preferred methodology and the standard of care for detecting genome-wide CNVs in a clinical lab [4]. CNV detection using ES is not currently a routine clinical test, likely due to the overwhelming inconsistencies among different methods [5–7] and the lack of a high-quality reference for CNVs from ES data. Most of the algorithms for CNV detection from ES data use the depth of coverage of exome targets under the assumption that the read depth is linearly correlated with the underlying true copy number at any given locus. However, the read depth in ES is known to be extremely variable and influenced by several factors such as sample batching, GC content, PCR duplication bias, targeted depth, sequencing efficiency, and mappability [8, 9]. These factors make it difficult to differentiate between technical artifacts and the real signal for a true copy number change. Also, detecting CNVs in polymorphic regions of the genome is challenging as the methods for ES-based CNV are estimating the copy number relative to the average copy number of the control samples.

While the sensitivity of several software tools for ES-based CNV has been published, reports of false discovery rate and the reproducibility are limited [7, 10]. Quality and performance standards for a clinical pipeline are set at the highest level possible as it has direct implications on patients’ health and disease management. In spite of the availability of several computational tools to detect CNVs from ES [11–14], clinical labs have been slow to adopt the incorporation of CNV detection from ES; however, some recent reports support the argument for copy number detection from ES in a clinical setting [15–17].

**Fig. 1:**
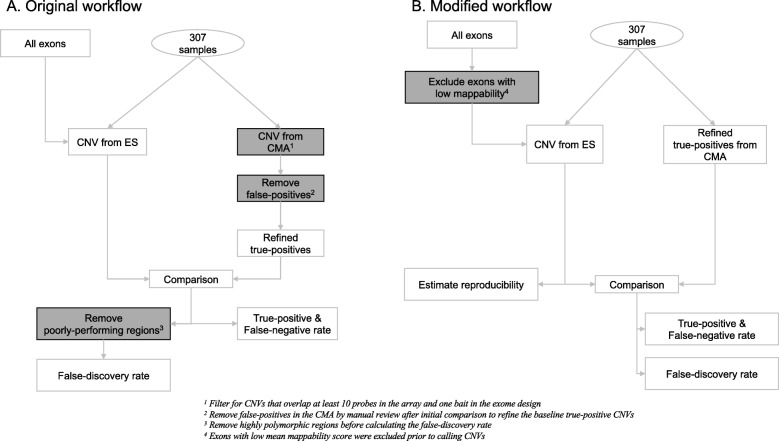
The default (**a**) and the modified (**b**) exome-based CNV detection and validation workflow

In this work, we used a cohort of 307 samples with clinical SNP array and ES data to create a dataset of high-quality true-positive CNVs from SNP array and comprehensively characterize the CNVs identified from ES data by assessing the false discovery rate, false negatives, and reproducibility. In addition, we proposed a modified analysis workflow to reduce false positives originating from the repetitive regions of the genome. We created a large-scale iterative variant calling framework using random control cohorts to assess the reproducibility (Fig. 1). Our results show that ES data can be used reliably for detecting clinically relevant CNVs with high sensitivity in a reproducible manner for use in clinical diagnostic settings.

## Methods

### CNV from exome sequencing

**Fig. 2:**
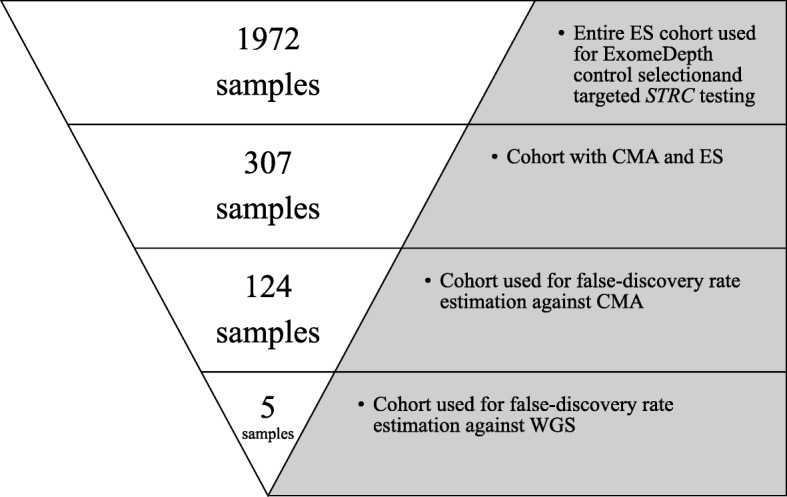
Schematic showing the cohorts used in this study

A cohort of 1972 individuals who were referred to the Genomic Diagnostic Laboratory (GDL) at the Children’s Hospital of Philadelphia (CHOP), Philadelphia, PA, for genetic testing and had ES data was collected for this study. All of the ES data were produced using the Agilent SureSelect V5 plus target capture kit (Agilent Technologies, Santa Clara, CA), and the aligned BAM files (*GRCh37*) were produced using the workflow described elsewhere [18]. All of the ES data were generated at the same sequencing center in 112 different batches over 2 years. These samples were analyzed as part of a process improvement program within the GDL at CHOP. Figure 2 shows the cohorts and the number of samples used in this study.

We used a custom CNV detection pipeline based on the R package ExomeDepth [19] with the default parameters and exon definitions provided along with the package. ExomeDepth creates a custom reference panel by choosing a subset of the most correlated samples (typically 10–12) from a larger control cohort to identify copy-number variants. Further details of this method are described elsewhere [19]. An initial cohort of 312 samples was selected from the 1972 samples with ES data that also had SNP array data. ExomeDepth recommends a high correlation value of 0.97 between the test sample and the reference panel for reliable results, and we excluded 5 samples based on poor correlation (*r*^2^ < 0.97). The final sample size for the comparison against the SNP array was 307 (166 males and 141 females), including 286 affected probands and 21 family members (16 unaffected and 5 affected). The mean number of CNVs per sample identified in the entire cohort was 145 with the default ES pipeline, with a standard deviation of 25. For each test sample, the rest of the cohort of 1971 samples was used as the pool of controls for ExomeDepth during the variant calling process. Summary statistics of the CNVs identified from ES are provided in Table 1.

**Table 1:** Characteristics of all the CNVs from ES with the default ExomeDepth workflow (307 samples)

	Deletions	Duplications
Number of CNVs	24,628	19,976
Number of exons	1 to 470 (mean = 4 (13 kb), median = 2 (968 bp))	1 to 981 (mean = 4 (14 kb), median = 2 (1.4 kb))
Number of CNVs per individual	45 to 210 (mean = 80, median = 78)	34 to 158 (mean = 65, median = 63)

### CNVs from SNP arrays

SNP array data for the abovementioned 307 samples were generated using the Illumina CytoSNP 850k chip. Poorly performing probes with poor cluster separation, probes with more than 3 genotype clusters, and probes within highly polymorphic CNV regions (> 10% internal cohort) were removed prior to CNV calling. CNVs from SNP arrays were called using CNV Workshop [20] or PennCNV [21]. CNVs involving fewer than 10 probes were excluded, as the rate of false positives increases with a fewer number of probes [22]. We excluded any CNVs called in the Y chromosome due to the lack of coverage on both the array and exome capture.

### Defining the high-quality true-positive CNVs from SNP arrays

We considered several factors while defining the high-quality baseline true-positive dataset for CNVs from SNP arrays, as it dictates the resulting sensitivity of the ES. In order to perform a reasonable comparison between these platforms, we first limited the CNVs from the arrays to have at least one coding exon and at least one bait in the ES design, and overlap at least ten SNP probes in order to minimize the number of false positives and false negatives from the SNP array dataset. In cases where we detected a CNV in the array but not in the ES, we manually reviewed the raw data from SNP array to determine if the call was a false negative in the ES or a false positive in the SNP array in order to refine the baseline true-positive calls as failing to remove the putative false positives from the SNP array would deflate the actual sensitivity of the ES.

In our validation cohort of 307 samples with the SNP array data, there were a total of 6634 CNVs before applying the exclusion criteria. After applying the filters, there were 487 CNVs (448 in the autosomes and 39 in chromosome X) for the initial true-positive dataset for comparison against the CNV calls from ES data. Thirty-two of these CNVs were not detected in the ES data in the initial comparison. These discrepant calls were manually reviewed using the Log_2_R ratio, B allele frequency, and the overall quality of the SNP probe clusters (e.g., cluster separation, normalized theta and normalized *R* values). Of these putative ES false positives, 24 were confirmed to be true positives in the array, 5 were false positives in the array, and 3 were ambiguous. The 8 CNVs that were either not present or ambiguous in the SNP array data were excluded, and the revised baseline true-positive dataset contained 479 CNVs (441 autosomal and 38 chromosome X variants). Details of this filtering cascade are provided in Table 2, and the summary statistics of the baseline true-positive CNVs are provided in Table 3. The final list of all the true-positive CNVs is provided in Additional file 1: Table S1.

**Table 2:** Filtering cascade to create a list of high-quality true-positive CNVs from the SNP arrays

Filtering cascade	Autosomal				Chromosome X					Total
	Loss		Gain		Loss			Gain		
	Het	Hom	Dup	Trip	Het	Hom	Hemi	Dup	Trip	
All	5166	194	1062	7	103	5	12	84	1	6634
CNVs with ≥ 10 probes	527	11	411	6	17	3	2	35	1	1013
CNVs with an overlapping exon and a bait	170	6	268	4	8	0	2	28	1	487
Refined true positives	165	6	266	4	7	0	2	28	1	479

*Het* heterozygous, *Hom* homozygous, *Hemi* hemizygous, *Dup* duplication, *Trip* triplication

**Table 3:** Characteristics of the high-quality true-positive CNV dataset as defined by the SNP array

	Characteristics of the baseline truth CNVs defined by the SNP array	
	Deletion	Duplication
Number of CNVs	180	299
Size	2.1 kb to 3.1 Mb (mean = 160 kb, median = 57 kb)	6 kb to 1.8 Mb (mean = 202 kb, median = 94 kb)
No. of SNP probes	10 to 2258 (mean = 67, median = 20)	10 to 774 (mean = 62, median = 29)
No. of exons overlapping the CNV	1 to 464 (mean = 15, median = 9)	1 to 359 (mean = 19, median = 11)
Small CNVs (< 4 exons)	36 (20%)	68 (23%)
Clinically reported CNVs	24 (13%)	17 (6%)

### Modification of the default ExomeDepth workflow

We used the 35-mer mappability score [23] from the UCSC genome browser [24] to compute mean mappability across each exon and excluded any exon with a mean mappability score less than or equal to 0.75 prior to computing the coverage and the variant calling. This threshold roughly corresponds to retain only the unique regions in the exome and resulted in the exclusion of 8527 (4.5%) out of the total 190,340 unique exons genome-wide which included 1132 exons (0.6%) that may be clinically relevant (Additional file 1: Table S2).

### Estimating false discovery rate of CNV from ES

To estimate the initial false discovery rate of the ExomeDepth pipeline, we reviewed CNVs called from ES data from the most recent 124 samples from the larger cohort of 307 samples. From these samples, there were 385 CNVs from the ES data that overlapped at least 10 probes in the SNP array design. Forty-two percent of these calls originated from 2 known extremely polymorphic regions, the killer cell immunoglobulin-like receptor region in chr19 (chr19:55,236,714-55,367,367) and the HLA region in chr6 (chr6:32,549,335-32,709,302). We did not review the CNVs from these 2 regions as they are known to be highly polymorphic and challenging in both platforms. The remaining 225 CNVs were compared to the SNP array data. Of these, 103 were also identified by the SNP array pipeline, while 122 were not identified by the SNP array pipeline but were manually reviewed by inspecting the Log_2_R ratio, B allele frequency, and genotype clustering of all the SNP array probes overlapping the CNV.

After the modification of the workflow, we followed the same protocol outlined above to estimate the false discovery rate in the 124 samples mentioned above. There were 249 CNVs from the ES data that overlapped at least 10 probes in the SNP array design. One hundred of these CNVs originated from the abovementioned polymorphic regions and were not reviewed. Eighty-four of the remaining CNVs were detected by the SNP array, and 65 were manually reviewed in the SNP array data by inspecting the Log_2_R ratio, B allele frequency, and genotype clustering of all the SNP array probes overlapping the CNV.

### Reproducibility of the ExomeDepth pipeline

To test the effect of the control cohort on the reproducibility of the CNVs identified from the ES pipeline (Table 1), we ran 1000 iterations of our pipeline for each of the 307 samples, using random subsets of 200 controls chosen from the remaining 1971 samples from the large ES cohort. We counted the number of times each CNV region was identified.

### Comparison against the whole-genome sequencing data

Paired-end WGS data (2 × 150 bp) for a small subset of 5 samples (5 out of the 307) was produced at the Broad Institute following the standard protocols for PCR-free WGS with an average coverage of 40×. Raw BAM (hg38) files were downloaded from the sequencing center and used for manual review of CNVs identified in ES. These 5 individuals were enrolled under an IRB-approved research protocol (CHOP IRB# 16-013231). In these 5 samples, we identified a total of 249 CNVs from WES data using the modified workflow (Additional file 1: Table S3), including 130 deletions and 119 duplications. Of these, 55 were removed for validation against the WGS data as they were inconsistently identified as both deletions and duplications over the 1000 iterations, likely due to the polymorphic nature and/or reference issues within the CNV region. The final sample size for validation against the WGS was 194 CNVs. Genomic coordinates for the CNVs detected from ES were in GRCh37 and were converted to hg38 using the software tool *liftover* [25]. Every CNV was deemed a true positive if the following evidence types were observed in the WGS data: (1) read depth across the called CNV compared to the regions flanking the breakpoints was consistent with the expected copy number state, (2) abnormal read pairs with larger than expected insert sizes, and (3) abnormal reads with soft-clipping and/or split mapping across the breakpoints. CNVs were marked false positive in the exome if none of these types of evidence was present in the WGS data or unsure if only some of the evidence types were present.

### Validation of CNVs using orthogonal methods

All diagnostic CNVs (*n* = 4) and a subset of 30 CNVs in the *STRC* gene were chosen for confirmation using PCR across the breakpoints, droplet digital PCR (ddPCR), or long-range PCR, using standard protocols used in the clinical laboratory [26]. The clinical validation protocol for *STRC* ddPCR is consistent with previously published studies [27].

## Results

### Default ExomeDepth workflow

Using a cohort of 307 samples, we validated an exome-based CNV detection pipeline using the R package ExomeDepth [19] for use in a clinical setting, by comparing the results against data from a high-quality set of true-positive CNVs from SNP array. The final dataset from SNP array comprised of 479 CNVs including 180 deletions and 299 duplications (Table 3). Of these, 36 of the deletions (20%) and 68 of the duplications (23%) overlapped fewer than 4 exons and were considered small CNVs. Compared to the SNP array, the default ES pipeline had a 96% true-positive rate for deletions and 95% for duplications. The default pipeline was 86% sensitive for the small deletions and 87% for the small duplications. A summary of the sensitivity rates from the default workflow for various CNV classes is provided in Table 4. There were 32 CNVs from the SNP array that were not identified by our exome pipeline. Twenty of the 32 false negatives were in highly polymorphic or segmental duplicated regions in the areas of the genome with no clinical significance. Fourteen false-negative CNVs involved first or last exons. Details of the CNVs missed by the exome pipeline are provided in Additional file 1: Table S4.

**Table 4:** Sensitivity of the default and modified ExomeDepth workflow

True-positive rate	Default ExomeDepth workflow		Modified ExomeDepth workflow	
	Deletions	Duplications	Deletions	Duplications
Overall	96% (172/180)	95% (283/299)	98% (163/166)	96% (280/293)
Heterozygous deletions	95% (164/172)		98% (157/160)	
Homozygous deletions	100% (6/6)		100% (4/4)	
Hemizygous deletions	100% (2/2)		100% (2/2)	
Duplications		95% (278/294)		95% (275/288)
Triplications		100% (5/5)		100% (5/5)
Autosomal	96% (165/171)	95% (256/270)	98% (156/159)	95% (254/266)
Chromosome X	78% (7/9)	93% (27/29)	100% (7/7)	96% (26/27)
Clinically reported CNVs	100% (24/24)	100% (17/17)	100% (22/22)	100% (17/17)
CNVs overlapping < 4 exons	86% (31/36)	87% (59/68)	94% (29/31)	87% (58/67)
CNVs overlapping ≥ 4 exons	98% (141/144)	97% (224/231)	99% (134/135)	98% (222/226)

In order to determine the false discovery rate from ES, we analyzed CNVs identified by the ES pipeline that overlapped at least 10 probes in the SNP array that should have been theoretically identified by the SNP array. Of the 225 ES variants identified in 124 selected samples, 103 were identified by SNP array and 23 were determined to be real in the SNP array after a manual review leaving 99 false positives accounting for a 44% false discovery rate in the ES data. Details of the CNVs identified from the exomes and not by the SNP array are provided in Additional file 1: Table S5.

### Modified ExomeDepth workflow

**Fig. 3:**
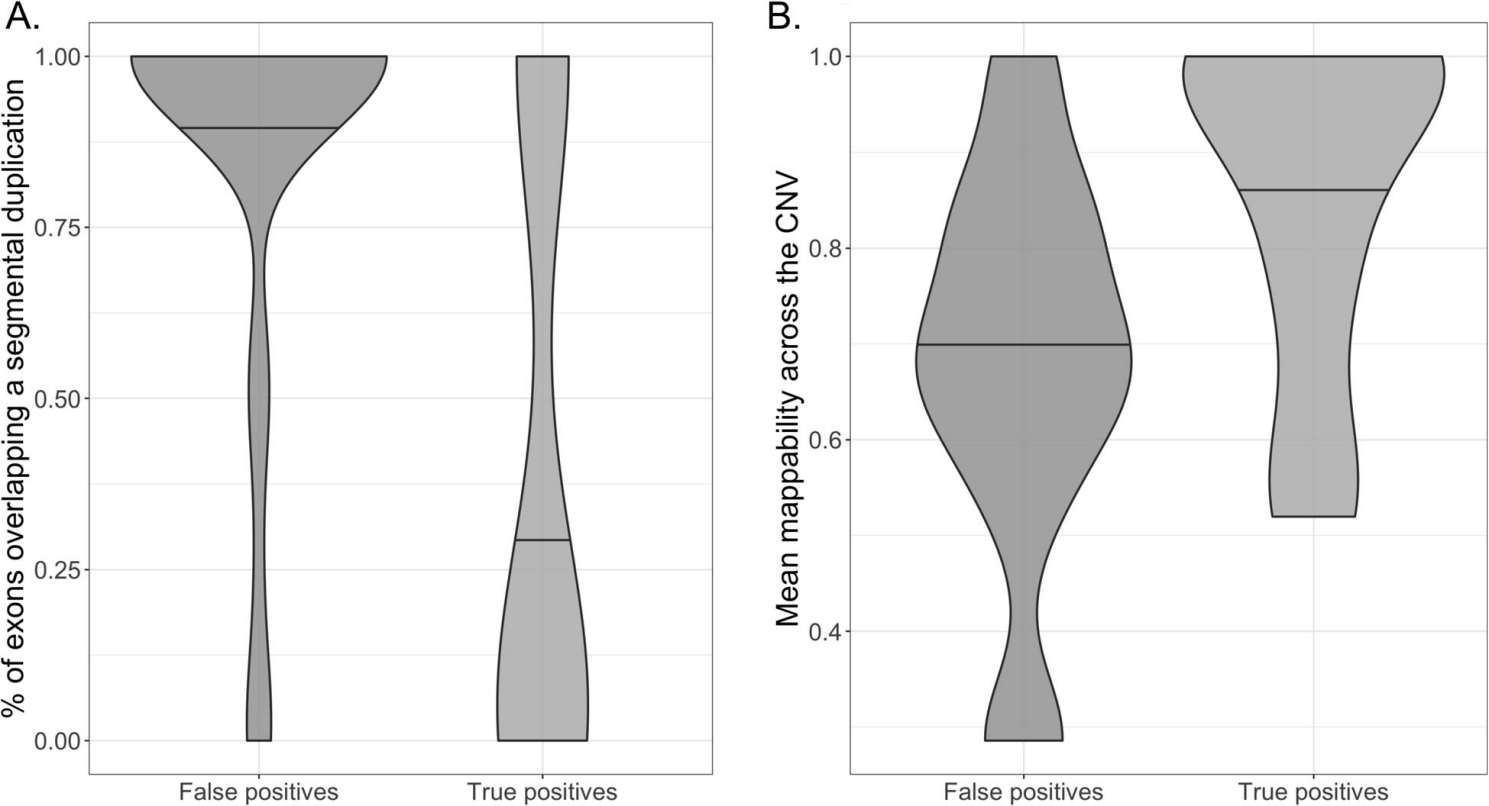
Analysis of CNVs from the false discovery rate cohort, stratified by false positives and true positives. **a** Violin plot of the percentage of exons that overlap segmental duplications within each CNV. **b** Violin plot of the mean mappability score across each CNV

Manual review of the false-positive CNV calls from ES data suggested that a large number overlapped with regions with high homology elsewhere in the genome and/or regions with low sequence complexity (Fig. 3a). Analysis of the mean mappability scores of the CNVs from the false discovery rate cohort showed that the false-positive CNVs had lower mean mappability scores compared to the true-positive CNVs (Fig. 3b). To mitigate this effect, we used a mappability filter to exclude exons that have difficulty in mapping short-read NGS data [28] prior to read counting and CNV calling.

**Fig. 4:**
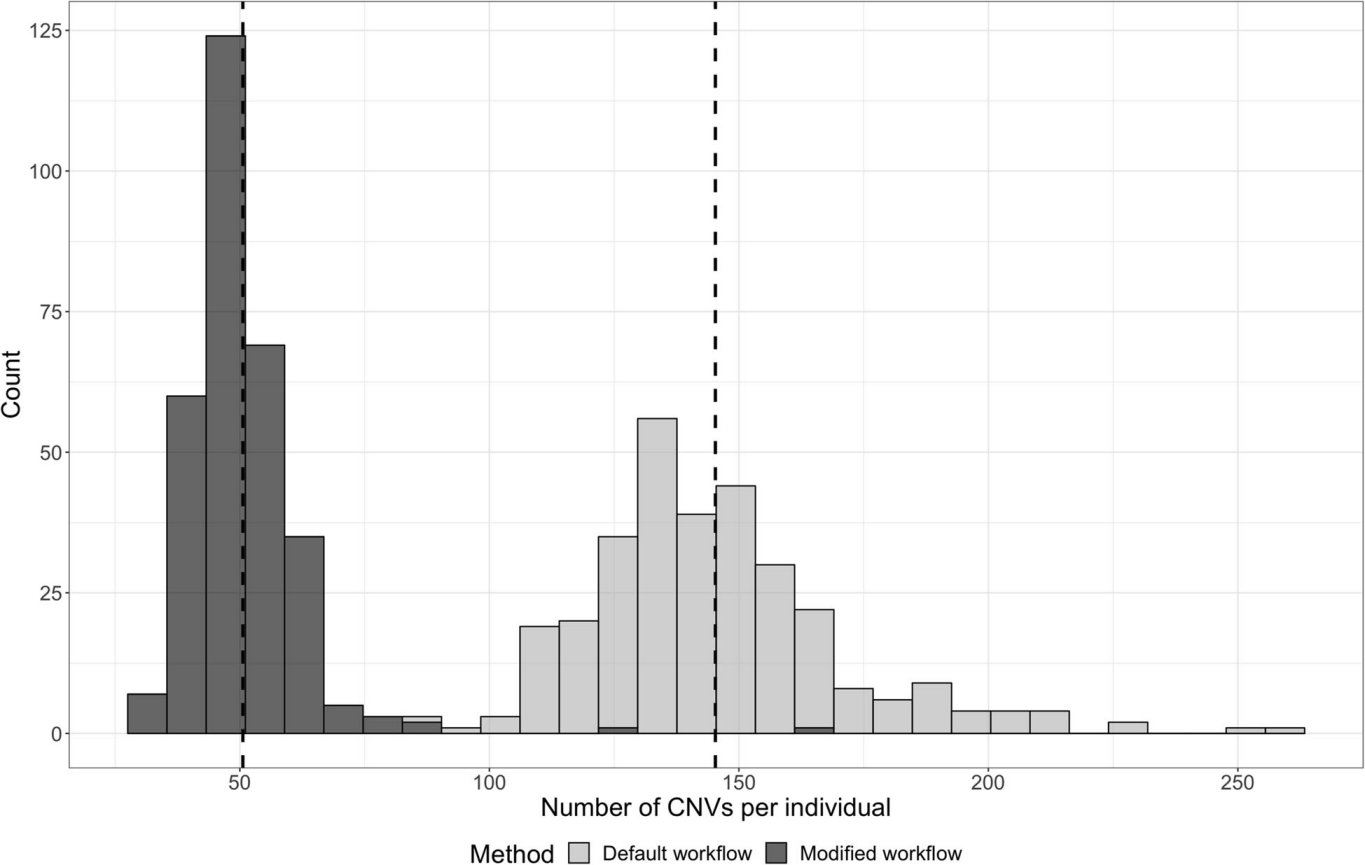
Histogram of the number of CNVs identified per individual using the default and the modified ExomeDepth workflow. The dotted lines represent the mean value for each group (51, 145 respectively)

With the mappability filter in place, the average number of calls identified reduced from 145 per sample (range of 83 to 259, with a median of 142) with the default workflow to 51 per sample (range of 31 to 167, with a median of 49). The distribution of the number of CNVs identified by the default and the modified workflow is shown in Fig. 4. Exclusion of exons with low mean mappability excluded 20 CNVs from the 479 high-quality CNVs resulting in 459 CNVs for further comparison against the modified workflow (Additional file 1: Table S1). Of the 20 excluded CNVs, only 2 were clinically reported and overlapped *HBA1/HBA2* (chr16:223477-227391), which are known to have high homology and low mappability. Compared to the true-positive calls (*n* = 459), the modified workflow had a higher sensitivity of 98% for the deletions and a slightly lower sensitivity of 96% for the duplications.

We repeated the protocol to estimate the false discovery rate by reviewing 149 CNVs and found that 17 of them were false positives in the ES data leaving the false discovery rate at 11.4%. The sensitivity of the modified ExomeDepth workflow is provided in Table 4.

To quantify the effect of the mappability filter on CNVs identified from highly homologous regions of the genome, we ran this modified workflow on a larger cohort of 1972 samples with ES data. Further, we chose to focus on the *STRC* gene, as it is one of the most common causes of autosomal recessive non-syndromic hearing loss and is often included in NGS-based panel testing. The *STRC* gene has a pseudogene that is 99.6% identical to the protein-coding gene, with the first 15 of the 29 exons identical between the 2 genes. We excluded the exons of *STRC* with a mappability score less than 0.75, leaving 3 exons in the modified workflow. With the original workflow, there were 953 *STRC* CNVs in 731 individuals (37% of the samples) and 147 individuals had both deletion and duplication identified in *STRC*. With the modified workflow, we identified a total of 76 *STRC* CNVs (29 deletions and 47 duplications) in 76 individuals (4% of the samples). Of these, we performed confirmatory testing on 6 duplications and 27 deletions involving all multiple exons, and 2 single-exon deletions. We were able to confirm all multi-exonic deletions and duplications, and the single-exon deletions were found to be associated with gene conversion events involving exon 24 and exon 26. Details of the validation of the *STRC* CNVs are provided in Table 5.

**Table 5:** Details of the validation of the CNVs identified in *STRC*

Genomic coordinates of the CNV from ES (hg19)	Exome call	Validation method	Result
chr15:43891026-43895609	Deletion	SNP array (*n* = 1), ddPCR (*n* = 2), long-range PCR followed by NGS (*n* = 1)	Heterozygous deletion of *STRC*
chr15:43891026-43940259	Deletion	SNP array (*n* = 2), ddPCR (*n* = 21)	Heterozygous deletion of *CATSPER2* and *STRC*
chr15:43891026-43940259	Duplication	ddPCR (*n* = 6)	Duplication of *CATSPER2* and *STRC*
chr15:43892733-43892880	Deletion	long-range PCR followed by NGS (*n* = 1)	Gene conversion involving exon 26 of *STRC*
chr15:43893595-43893749	Deletion	long-range PCR followed by NGS (*n* = 1)	Gene conversion involving exon 24 of *STRC*

### Reproducibility

To understand the effect of the choice of the control cohort, we estimated the reproducibility of our pipeline by rerunning every sample for 1000 iterations using a pool of 200 randomly selected controls and counted the number of iterations where the initial CNVs were also detected. Over these 307,000 experiments, the average number of CNVs per sample across iterations was 60 for the autosomes (median = 50) and 2 for the X chromosome (median = 1.4). There was a total of 3787 out of 13,804 CNVs (27%) in the cohort which were identified in all 1000 iterations, with a mean of 12 CNVs per individual. All the clinically reported variants from the SNP array (*n* = 39) and 4 new diagnostic CNVs from the ES were identified in all 1000 experiments for those samples.

As most of the CNVs identified by the ES are below the resolution of SNP array, we performed a secondary validation using the whole-genome sequencing data from 5 samples with both exome and genome data. Of the 194 CNVs identified from ES data in these 5 samples, 43 CNVs were called in all 1000 iterations (22%). When compared to the WGS data, 56% (24/43) were considered to be true positives leaving the false discovery rate at 44%, while the remaining CNVs had no supporting or ambiguous reads in the WGS data. When the threshold for the number of reproducible iterations was reduced to 900, the false discovery rate increased to 64% (67/106), and for a threshold of 800 iterations, the false discovery rate increased further to 68% (87/129). Overall, the 42 true-positive CNVs had a mean number of iterations of 987 (range, 854–1000; median, 1000). Using the minimal number of iterations for the known true positive (854), there were 28 CNVs per individual on average compared to only 12 variants per individual identified in all 1000 iterations. The majority of the false-positive CNVs were found to be associated with either segmental duplications overlapping the exons that escaped the mappability threshold, the presence of multiple haplotypes, or polymorphic regions (Additional file 1: Table S3).

### New diagnoses

Upon reviewing CNVs which overlapped known disease genes, we identified four new diagnostic CNVs in four individuals who had non-diagnostic results with the SNV/indel only exome pipeline and the SNP array. We were able to determine the exact breakpoints for two deletions from the chimeric reads in the exome data and validated these using Sanger sequencing across the breakpoints. The details of the new diagnoses are provided in Table 6, and brief phenotype descriptions are provided in Additional file 2. These pathogenic CNVs involved two to five exons, in both autosomal dominant and recessive disease-associated genes. Table 7 provides the number of CNVs identified by the default ExomeDepth workflow, the modified workflow, and filtering cascade with the number of reproducible CNVs and the number of clinically relevant CNVs for these individuals.

**Table 6:** New diagnoses made by the ES pipeline that were previously not reported

ID	Genomic coordinates (hg19)	Gene	Size (bp)	CNV	Number of exons	SNPs in SNP array	Comment	Confirmation method
1	chr12:116,457,030-116,460,406	*MED13L*	3376	Het del	3	3	Under SNP array resolution	ddPCR
2	chr6:33,405,980-33,409,266	*SYNGAP1*	3286	Het del	5	4	Under SNP array resolution	ddPCR
3	chr3:191,888,248-192,126,012	*FGF12*	237,765	Dup	4	123	Not known disease gene	SNP array and breakpoint sequencing
4	chr4:123,976,639-123,989,201	*SPATA5*	12,562	Het del	2	3	Under SNP array resolution, in trans with SNV	ddPCR and breakpoint sequencing

**Table 7:** Number of CNVs identified by the modified ExomeDepth pipeline at every stage

ID	Number of CNVs identified by the default pipeline	Number of CNVs identified by the modified pipeline	Number of reproducible CNVs (> 850 iterations)	Number of CNVs in OMIM disease genes	Number of diagnostic CNVs relevant to the patient phenotype
1	137	47	27	3	1
2	152	54	33	1	1
3	163	54	26	1	1
4	174	53	26	1	1

## Discussion

Detecting CNVs from ES data is perceived as challenging for clinical use as many previously published reports suggested high false-positive rates and low sensitivity. Several algorithms exist for detecting CNVs from ES and often there is a trade-off between the true-positive rate (detecting the true CNVs) and false discovery rate (detecting false positives). For use in a clinical setting, one requires the highest sensitivity and lowest false discovery rate possible for a given platform. In this work, we used a cohort of 307 individuals to systematically and comprehensively benchmark the ability to detect CNVs from exome sequencing data using the ExomeDepth pipeline. Prior benchmarking efforts have attempted to use data from multiple orthogonal platforms and algorithms for estimating sensitivity and specificity from ES data [7, 28, 29]. Performance metrics such as sensitivity and false discovery rate rely solely on the baseline true-positive set. Factors influencing such comparisons should be considered carefully before defining the true-positive set as the resulting performance metrics may be misleading. For example, in SNP array, the number of probes within a CNV is known to be associated with the confidence of the call and the rate of false positives increases with fewer number of probes [22]. Non-exon targeted, genome-wide arrays are considered to be low resolution for small CNV (< 4 exons) detection and may not be the best orthogonal technology for benchmarking against ES; however, SNP array is a standard of care test for the detection of clinically relevant CNVs genome-wide. Taking these limitations into account, we have created a high-quality baseline true-positive CNVs from SNP array data by iterative review process by manually verifying the B allele frequency, Log-*R* ratio, and the clustering quality of the probes underlying the copy-number variants. This process of manual review of SNP array data enabled us to have a bona fide true positives for further comparisons. The limitations of ES include poor sequencing efficiency in GC-rich regions, in segmental duplication regions, and in regions with low sequence complexity [8, 9]. Also, the sequencing is based on a target design which may or may not include regions of the genome that is captured by an alternative technology (SNP array in this case). Taking the inherent limitations of both the ES and SNP array platforms into account allowed for a high-quality true-positive CNV dataset for a fair comparison across platforms.

The ExomeDepth pipeline coupled with a mappability threshold for including exons before calling the CNVs reduced the number of calls to one third which reduced the burden of downstream analysis and validation. In addition, we were able to detect CNVs in the clinically relevant and difficult regions, such as *STRC*, with a 100% validation rate in the samples tested. The current standard of care testing for the *STRC* gene is a ddPCR assay of exon 23 and intron 25 [27]. Our work includes a signal from 3 exons (exons 23, 24, and 26) which is an improvement considering the current standards. We found that our modified ES workflow is 97% sensitive for both deletions and duplications. In reviewing the false negatives, the majority involved polymorphic or segmental duplication regions and the first or last exon of a gene. First exons are known to be GC rich, and GC content greatly influences the depth of coverage [11]. However, there is no prior evidence associating the last coding exons with a non-uniform depth of coverage during exome sequencing. The modified pipeline had a false discovery rate of 11.4% compared to the standard of care SNP array. The analysis of false positives showed a similar trend regarding polymorphic, low sequence complexity, or segmental duplications with 89% located in these regions, with the remainder being CNVs involving the first/last exon of 2 nearby genes. The results from the ExomeDepth pipeline were 100% reproducible for clinically reported variants with control datasets generated from over 112 batches over a period of 2 years, even though sample batching is known to be strongly correlated with the variability in the depth of coverage observed in ES data [11].

Using this pipeline, we were able to make new diagnoses in four individuals who had previous negative SNP array tests (additional diagnostic yield 4/286 = 1.4%), demonstrating the utility of this assay for small (two to five exon CNVs), intragenic CNVs below the clinical reporting threshold for SNP array.

Despite our positive results, some challenges still exist, for example, determining the exact copy number state in highly variable regions of the genome regardless of the technology used. Detecting CNVs in genes with near-identical homologs elsewhere in the genome is an intractable problem when using short-read sequencing data. These regions often result in both false positive and false negative CNV calls. Using an exon-level mean mappability score threshold helps in reducing the false positives, but it also excludes some clinically relevant genes completely (e.g., *SMN1* and *SMN2*). In our experiments, using the mappability filter excluded two clinically reported variants in the SNP array overlapping *HBA1/HBA2*. These genes are difficult to assay by short-read sequencing as they overlap segmental duplications. Averaging the mappability scores across exons allows the inclusion of exons with partial regions of poor mappability, resulting in numerous false positives. The 100% validation rate we observed for the *STRC* gene deletions and duplications after using the mappability-based filter is encouraging, and further work is warranted to understand the effect on similar regions elsewhere in the genome.

In spite of being able to detect the clinically reported CNVs 100% of the time in our reproducibility experiments, we found that the choice of controls and the batch in which the controls were sequenced had an effect on the total number of CNVs identified. While the number of times a variant is reproduced with different control cohorts may indicate the robustness of the call, it is also likely that technical artifacts are also reproducible. It is important to note that all of our controls were generated in the same sequencing facility with the same protocol. Also, the number of control samples available for the iterative variant calling process may limit the ability to conduct such a large-scale experiment. Further work is warranted to utilize the reproducibility along with other quality metrics for better ranking of likely true-positive variants. Validation of clinically relevant CNVs using an orthogonal method is important before reporting to the patients as ES-based CNV detection is still relative to the control cohort used. However, maintaining good practices in creating a control cohort, a validation pipeline with a variety of known variant types and size, and stringent quality control before clinical correlation will reduce the burden of validations using orthogonal methods.

Based on the results presented in this paper, we have a few considerations for the detection of CNVs from exomes with high sensitivity and how to prioritize high-quality CNVs for the identification of clinically relevant CNVs. We have shown that the CNVs identified and the reproducibility depend on the choice of controls, and it is important to keep the control cohorts updated in a frequent manner. It is also important to make sure the controls are produced using the same sequencing platform, library preparation methods, and target capture kits. We recommend excluding exons with low mean mappability prior to variant calling as these regions tend to be challenging for the alignment of short reads, and the results from these regions are not reliable. A large-scale iterative variant calling process with random controls can help assess the reproducibility of the CNVs identified in the initial call set, and the number of iterations in which a particular variant is identified can be used to rank the variants in terms of reliability. Together, these measures reduce the total number of variants per individual. Annotations such as overlap with segmental duplications and alternative haplotypes will help further reduce the number of variants for downstream analysis as they are more likely to be enriched for false positives and less reliable. Finally, we highly recommend the validation of the CNVs by an orthogonal method such has ddPCR or quantitative PCR (qPCR) before reporting to the patients.

## Conclusions

In summary, our work demonstrates the ability to detect CNVs from ES data in a reliable and reproducible manner in a clinical setting. Integrating CNVs in a clinical workflow may help in finding molecular diagnoses for unresolved patients with one pathogenic variant (SNV/indel) in an autosomal recessive disease gene and increase the overall diagnostic rate. We expect targeted NGS to be used in diagnostics for a considerable amount of time given the lower cost, focused approach, and reduced burden on downstream analysis compared to genome sequencing. Thus, it is important to continue to invest in resources and refine the existing tools for making ES a better diagnostic test overall.

## Supplementary information

**Additional file 1: Table S1.** Details of the high-quality true-positive CNVs from SNP array and the exome validation status. **Table S2.** List of exons in disease-associated genes that were excluded based on a mean mappabilty score of 0.75 or less. **Table S3.** Details of the CNVs used for false-discovery rate estimation against WGS data. **Table S4.** Details of the ES false negatives and SNP array false positive CNVs. **Table S5.** Details of CNVs that overlapped 10 SNPs in the SNP array and reviewed for determining false-discovery rate against SNP array.

**Additional file 2:** Phenotype information for the patients with diagnostic CNVs.

## Data Availability

The ES and SNP array datasets generated and analyzed during the current study are not publicly available as they are patient samples tested in a clinical diagnostic lab and sharing them could compromise research participant privacy. The five patient samples with WGS data may be made available upon reasonable request. The following public databases and open software were used: ExomeDepth (https://cran.r-project.org/web/packages/ExomeDepth/index.html) [30] Encode Mappability Uniqueness (35 bp) Scores from UCSC database (http://hgdownload.cse.ucsc.edu/goldenPath/hg19/encodeDCC/wgEncodeMapability/wgEncodeDukeMapabilityUniqueness35bp.bigWig) [24] UCSC LiftOver (https://genome-store.ucsc.edu/) [31]
