# Supplementary material for: A highly sensitive and specific workflow for detecting rare copy-number variants from exome sequencing data
Source: Genome Med. 2020 Jan 30;12:14. doi: 10.1186/s13073-020-0712-0 (PMC6993336; doi:10.1186/s13073-020-0712-0)
Supplement: Supplementary file 2 — Additional file 2: Phenotype information for the patients with diagnostic CNVs. [file 13073_2020_712_MOESM2_ESM.docx]

**Additional File 2.**

**A highly sensitive and specific workflow for detecting rare copy-number variants from exome sequencing data**

Ramakrishnan Rajagopalan MS^1,2^, Jill Murrell PhD^1,3^, Minjie Luo PhD^1,3^, Laura K. Conlin PhD^1,3*^

*^1^Division of Genomic Diagnostics, Dept. of Pathology, Children’s Hospital of Philadelphia, PA.*

*^2^School of Biomedical Engineering, Science and Health Systems, Drexel University, Philadelphia, PA.*

*^3^Department of Pathology and Laboratory Medicine, Perelman School of Medicine, University of Pennsylvania, Philadelphia, PA.*

**New diagnoses in known autosomal dominant disease genes**

### Patient 1

Patient 1 presented with global developmental delay, dysmorphic features, speech regression, generalized hypotonia, esotropia, autistic features and partial agenesis of the corpus callosum. Clinical ES test did not provide a molecular diagnosis. Our pipeline identified this *de novo* deletion in the gene *MED13L* (exons 5-7) which was confirmed with ddPCR. A retrospective analysis of the SNP array data revealed a 3 SNP deletion which was not originally reported as it did not meet clinical reporting criteria.

### Patient 2

Patient 2 presented with moderate global developmental delay, seizures, severe myopia, strabismus, hypotonia and feeding difficulties. Previous testing was included negative chromosomal SNP array and ES. The exome-based CNV pipeline identified a 3kb deletion in the *SYNGAP1* gene (exons 8-16). The heterozygous deletion of exon 10 was confirmed in the patient with ddPCR. This deletion was also seen in the SNP array but was under clinical reporting criteria.

### Patient 3

Patient 3 presented with severe refractory epileptic encephalopathy, developmental regression, spasticity, delayed skeletal maturation and cerebellar atrophy. The patient also had immunodeficiency, recurrent infections, anemia and unexplained fever. Previous genetic testing, chromosomal SNP array, ES, and mitochondrial gene panel. The CNV pipeline identified a 583kb duplication of the *FGF12* gene that included all but the last exon in all *FGF12* transcripts. This duplication was also detected in the SNP array but was not reported at the time of testing, as *FGF12* has been associated with human disease, including a similar duplication, after the time of clinical testing^1^. The duplication was confirmed in our proband and was absent in both his parents using PCR that spanned the duplication breakpoints followed by Sanger sequencing, as previously reported^1^.

## **New diagnosis in autosomal recessive gene *in trans* with sequencing variant**

### Patient 4

Patient 4 presented with bilateral sensorineural hearing loss, global developmental delay, nystagmus, strabismus, hypermetropia, feeding difficulties and axial hypotonia. The patient had a normal chromosome SNP array and an inconclusive ES test results, including a paternally inherited missense variant in *SPATA5*. The CNV pipeline identified a heterozygous two exon deletion in the gene SPATA5 (exons 12-13), which was found to be maternally inherited. Re-analysis of the SNP array identified the same deletion, which was too small to be clinically reported. Biallelic loss of function variants in *SPATA5* have been reported in patients with bilateral sensorineural hearing impairment, global developmental delay, nystagmus, strabismus, feeding difficulties, seizures, and axial hypotonia^2^. The heterozygous deletion of exon 12 was confirmed in the patient and her mother with ddPCR. Also, PCR was performed using primers outside the breakpoints followed by Sanger sequencing.

**References**

1 Shi, R. M. *et al.* Phenytoin-responsive epileptic encephalopathy with a tandem duplication involving FGF12. *Neurol Genet* **3**, e133, doi:10.1212/NXG.0000000000000133 (2017).

2 Buchert, R. *et al.* SPATA5 mutations cause a distinct autosomal recessive phenotype of intellectual disability, hypotonia and hearing loss. *Orphanet J Rare Dis* **11**, 130, doi:10.1186/s13023-016-0509-9 (2016).
